# A Novel *Ex Vivo* Isolation and Expansion Procedure for Chimeric Antigen Receptor Engrafted Human T Cells

**DOI:** 10.1371/journal.pone.0093745

**Published:** 2014-04-03

**Authors:** Marc Cartellieri, Stefanie Koristka, Claudia Arndt, Anja Feldmann, Slava Stamova, Malte von Bonin, Katrin Töpfer, Thomas Krüger, Mathias Geib, Irene Michalk, Achim Temme, Martin Bornhäuser, Dirk Lindemann, Gerhard Ehninger, Michael P. Bachmann

**Affiliations:** 1 Institute of Immunology, Medical Faculty ‘Carl Gustav Carus’, TU Dresden, Dresden, Germany; 2 Helmholtz Zentrum Dresden-Rossendorf, Institute of Radiopharmaceutical Cancer Research, Department of Radioimmunology, Dresden, Germany; 3 Medical Clinic and Polyclinic I, University Hospital ‘Carl Gustav Carus’, TU Dresden, Dresden, Germany; 4 Department of Neurosurgery, Section Experimental Neurosurgery and Tumor Immunology, Medical Faculty ‘Carl Gustav Carus’, TU Dresden, Dresden, Germany; 5 Institute of Virology, Medical Faculty ‘Carl Gustav Carus’, TU Dresden, Dresden, Germany; 6 Center for Regenerative Therapies Dresden, TU Dresden, Dresden, Germany; Copenhagen University Hospital at Herlev, Denmark

## Abstract

Genetically engineered T lymphocytes are a promising option for cancer therapy. Prior to adoptive transfer they have to be expanded *in* *vitro* to reach therapeutically sufficient numbers. So far, no universal method exists for selective *in vitro* expansion of engineered T lymphocytes. In order to overcome this problem and for proof of concept we incorporated a novel unique peptide sequence of ten amino acids as epitope (E-Tag) into the binding domains of two novel chimeric antigen receptors (ECARs) directed against either prostate stem cell antigen (PSCA) for the treatment of prostate cancer (PCa) or CD33 for the treatment of acute myeloide leukemia (AML). The epitope tag then was utilized for expanding ECAR engrafted T cells by triggering the modified T cells via a monoclonal antibody directed against the E-Tag (Emab). Moreover, the E-Tag served as an efficient selection epitope for immunomagnetic isolation of modified T cells to high purity. ECAR engrafted T cells were fully functional and mediated profound anti-tumor effects in the respective models of PCa or AML both *in vitro* and *in vivo*. The method can be integrated straightforward into clinical protocols to improve therapeutic efficiency of tumor treatment with CAR modified T lymphocytes.

## Introduction

Tumor treatment with genetically engineered T lymphocytes is currently a rapidly evolving field at the turning point from preclinical *in vitro* and *in vivo* experiments to clinical applications [1]–[3]. T lymphocytes are either armed with antigen-specific T cell receptors (TCRs) or chimeric antigen receptors (CARs) to render them tumor-specific. CARs combine the cellular and humoral arm of the immune response by assembling a binding moiety, which provides the antigen-specificity, and an activating immune receptor [4]. Commonly the antigen-binding moiety is a single-chain fragment variable (scFv) derived from a tumor-antigen-specific monoclonal antibody (mab). Once such artificial immune receptors are expressed at cell surfaces of genetically modified T lymphocytes, they can bind to their antigen and transmit an activating signal, which in turn triggers T cell effector functions against target cells. Engraftment with CARs enables T cells for MHC-independent antigen recognition, thus major immune escape mechanisms of tumors such as downregulation of MHC molecules are efficiently bypassed [5]. Furthermore, proliferation and survival of modified T cells can be improved by the implementation of a multitude of signaling domains from different immune receptors into a single CAR and thereby rendering T cells more resistant to the immunosuppressive milieu in tumor tissue [6]–[10]. In addition to cancer immunotherapy, CAR modified lymphocytes have been successfully applied for the treatment of virus infections [11], [12] and first experimental studies have been published using CARs engrafted onto regulatory T cells (Tregs) for the treatment of autoimmune diseases [13]–[15]. Recently, first clinical trials with second-generation CARs, which in addition to the activating CD3ζ chain harbor a costimulatory signaling sequence, have been undertaken and CAR engrafted T lymphocytes have proven to be highly efficient in eradicating leukemias of B cell origin [16]–[19]. However, with current methods only part of the polyclonal donor T cell population can be successfully genetically modified. Thus, a mixed population of unmodified non-specific and modified tumor-specific effector cells is generated inevitably. Furthermore, initial numbers of modified T lymphocytes have to be increased *in vitro* to obtain sufficient cells for treatment. Current protocols expand them either non-specifically with mitogenic αCD3 and αCD28 antibodies [20], [21], or make use of genetically modified antigen-presenting cell lines, which express the target antigen and in some cases additional costimulatory molecules [22], [23]. Whereas the first approach does not allow for enrichment of antigen-specific T lymphocytes and often results in decreased frequencies of antigen-specific T cells, the second approach is always restricted to a certain antigen and cannot be applied universally. Moreover, each batch of generated T lymphocytes might be contaminated with activator cells and therefore has to be tested before clinical application. The shortcomings of the currently available protocols prompted us to develop a method which allows expansion and purification of CAR modified T lymphocytes independent of their tumor antigen-specificity.

## Results

### The E-Tag can be Incorporated as Linker into the Binding Moiety of CARs without Disturbing their Functionality

**Figure 1:**
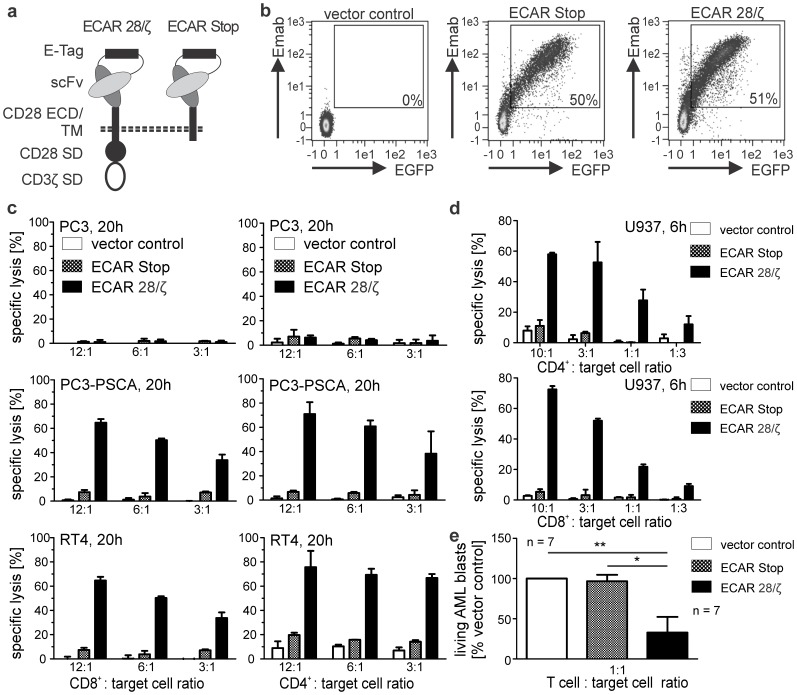
Incorporation of the E-Tag as linker in the binding moiety of second-generation CARs does not disturb their functionality. (**a**) For generation of ECARs a single-chain fragment variable (scFv) was fused to the CD28 molecule (aa 20–200) including the extracellular domain (ECD), transmembrane domain (TM) and intracellular signaling domain (SD). The CD3ζ chain (aa 55–164) was fused to the C-terminus of the CD28 signaling subunit separated by a (GGGGS)_4_-linker (ECAR 28/ζ). The variable light and heavy chains of the scFvs are connected by the E-Tag epitope flanked by a stretch of G_4_S_1_. As control a Stop variant of the signaling CAR (ECAR Stop) was generated lacking any intracellular sequences downstream of the CD28 transmembrane domain. (**b**) Isolated human T cells were genetically modified using lentiviral gene transfer and subsequently stained for the presence of ECARs on the cell surface using an E-Tag specific mab (Emab). An EGFP marker gene is co-translated in stably transduced cells and EGFP fluorescence can be used to distinguish ECAR modified from non-modified T lymphocytes. Percentages of ECAR and EGFP double positive T cells are given for each plot. (**c**) Human CD8^+^ and CD4^+^ T cells from healthy donors were engrafted with PSCA-specific ECARs and subsequently tested for their killing abilities against PSCA-negative (PC3) and PSCA expressing (PC3-PSCA, RT4) PCa cell lines in a ^51^Cr-release assay at indicated T cell to target cell ratios. One representative of four analyzed donors is shown. (**d**) Likewise, human CD8^+^ and CD4^+^ T cells were genetically modified with CD33-specific ECARs and subsequently tested for their killing abilities against the CD33 expressing AML-derived cell line U937 in a ^51^Cr-release assay at indicated T cell to target cell ratios. One representative of six analyzed donors is shown. (**e**) CD33-specific ECAR armed T cells from healthy donors were incubated with sorted eFluor670-labeled CD3^−^CD19^−^ blasts from AML patients (blast content >70%) at a total T cell to target cell ratio of 1∶1. After 48h the absolute number of living (PI-negative) eFlour670-positive cells was determined by flow cytometry using a MACSQuant Analyzer. The number of living cells was normalized to the vector control sample. Mean and SD of 7 different donor/patient pairs are shown.

Our approach is based on the incorporation of an epitope into the extracellular part of a CAR, which then could be utilized for selective engagement of CAR modified T cells via a mab specific for this epitope. Furthermore, we intended to use the epitope as a tag for isolation of engineered cells. The scFv providing the antigen-specificity is the most distant domain of a CAR from the cell membrane and hence should extrude the extensive glycocalyx, which covers the cell surface of eukaryotic cells [24]. Therefore, we reasoned that the incorporation of the epitope into a scFv linker should ensure easy access for binding of the epitope-specific mab. As an epitope we introduced a peptide of 10 amino acids (aa) length flanked by a single glycine-serine (G_4_S) stretch on both sides as a linker in between heavy and light chain of our scFvs (**Fig. 1a** **)**. The peptide termed E-Tag is derived from the human nuclear protein La/SS-B and has recently been described by our group together with the mab recognizing this peptide motif [25]. The resulting epitope-tagged CAR constructs were subsequently termed ECARs. The signaling chain of ECAR constructs was derived from the cytoplasmic domains of the human CD3ζ and CD28 molecule arranged in tandem to provide combined activating and costimulatory signal induction upon recognition of the respective antigen (**Fig. 1a**). For prove of principle, two scFvs recognizing two different tumor antigens were modified with the E-Tag linker. The modified scFvs recognize either prostate stem cell antigen (PSCA), a prostate cancer (PCa) specific antigen [26], [27] or CD33, a cell surface glycoprotein specific for hematopoietic cells of myeloid origin [28]. Binding affinities of the E-Tag modified scFvs were compared to respective scFvs in which the light and heavy chains were connected by a conventional G_4_S-linker instead of the E-Tag linker and found to be similar as shown for the PSCA-specific scFv in **Fig. S1a**. Both antigens are suitable targets for immunotherapy of PCa [29], [30] or acute myeloid leukemia (AML) [31]–[33], respectively. In all experiments an ECAR without any signaling domain (ECAR Stop) but otherwise similar to the functional ECAR was engrafted onto T lymphocytes as control (**Fig. 1a**).

First, we confirmed that ECARs are expressed on the cell surface of human T lymphocytes and the E-Tag is accessible for ab binding by staining the genetically modified T cells with the E-Tag specific mab (Emab, **Fig. 1b** **, Fig. S1b**). Next, we performed a series of *in vitro* experiments to verify that the epitope linker does not interfere with binding of the scFv to its antigen and hence functionality of the ECAR. Both human CD8^+^ and CD4^+^ T cells engrafted with a PSCA specific ECAR very efficiently killed PCa cell lines expressing the PSCA antigen, whereas antigen-negative prostate cells were not attacked (**Fig. 1c**). In a similar way, human T cells expressing an ECAR with CD33 specificity recognized and lysed CD33 expressing AML cells (**Fig. 1d**). In addition, human T cells equipped with ECARs secreted proinflammatory cytokines like IFN-γ and TNF as well as growth-promoting IL-2 and started to proliferate upon culture with antigen-expressing cells (**data not shown**). Moreover, we could proof, that T cells from healthy human donors armed with the CD33-specific ECAR were able to lyse patient derived AML blasts very efficiently (**Fig. 1e**).

Thus, we could demonstrate that the E-Tag can be incorporated into the linker region of two scFvs with different specificities without disturbing the binding to their respective antigens, signal transduction or activation of the ECAR engrafted T lymphocytes upon antigen recognition.

### ECAR Engrafted T Cells can be Activated and Expanded *in vitro* via the E-Tag

**Figure 2:**
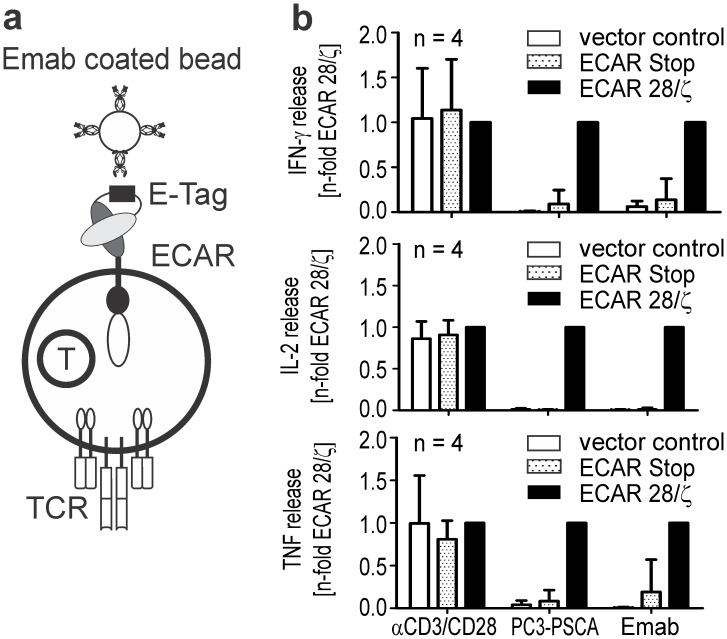
ECAR genetically modified human T cells can be activated via the E-Tag. (**a**) Principle of specific activation of ECAR engrafted T cells independent of their antigen specificity by adding Emab coated activator beads used as artificial APCs. (**b**) Secretion of IFN-γ, TNF and IL-2 from T cells engrafted with a PSCA-specific ECAR upon incubation with either antigen-positive target cells or Emab coated magnetic beads. For comparison, T lymphocytes were incubated with polyclonal αCD3/CD28 activator beads. The absolute amount of secreted cytokines was normalized to the ECAR 28/ζ sample and mean ± SD of 4 donors is shown.

**Figure 3:**
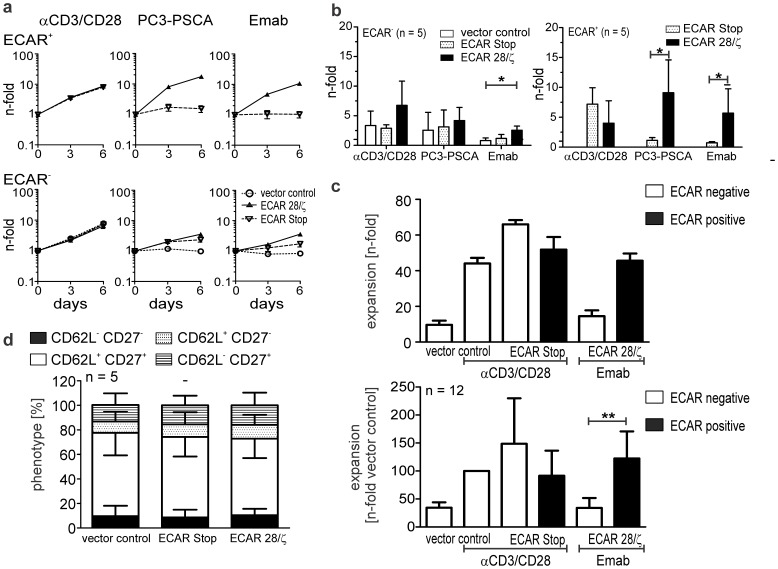
ECAR genetically modified human T cells can be specifically expanded with Emab coated magnetic beads to high numbers. Expansion of T lymphocytes engrafted with a PSCA-specific ECAR after activation with Emab coated magnetic beads. For comparison, genetically modified T lymphocytes were either incubated with antigen-positive target cells (PC3-PSCA) or polyclonal αCD3/CD28 activator beads in the absence of exogenous cytokines. Absolute cell numbers were determined at day 0, 3 and 6 using a MACSQuant Analyzer. ECAR+ T cells were distinguished from ECAR T cells by co-expression of the EGFP marker. Expansion of T lymphocytes off one representative donor over 6 days (**a**) and mean ± SD of 5 donors (**b**) are shown. Statistical analysis was performed using non-parametric one-way ANOVA (Kruskal-Wallis test) combined with post-hoc Dunn’s Multiple Comparison test or non-parametric Mann-Whitney test (*p<0.05). (**c**) T lymphocytes were engrafted with either PSCA- or CD33-specific ECARs and expanded for 12–14 days with Emab coated activator beads in the presence of recombinant cytokines as described in the Materials and Methods section. For comparison, T lymphocytes engrafted with vector control or PSCA−/CD33-specific ECAR Stop variants were expanded with αCD3/CD28 polyclonal activator beads. The expansion rates for one representative donor and mean ± SD of expansion rates normalized to the polyclonally stimulated vector control for 12 donors are shown. Statistical analysis was performed using non-parametric one-way ANOVA (Kruskal-Wallis test) and post-hoc Dunn’s Multiple Comparison test (**p<0.01). (**d**) Expanded T lymphocytes were stained with fluorochrome-labeled mabs directed against CD3, CD27 and CD62L surface antigens and analyzed using a MACSQuant Analyzer. Relative percentage of CD62L^+^ CD27^+^ double positive cells (white area), CD27^+^ (striped area) or CD62L^+^ (dotted area) single positive cells and double negative cells (black area) of Emab expanded ECAR engrafted T lymphocytes or control T lymphocytes expanded with polyclonal activator beads are shown. No significant differences could be detected.

Next, we evaluated if the E-Tag can in principle be used for specific activation of ECAR engrafted T cells independent of antigen-specificity of the artificial receptor. As artificial APCs commercially available magnetic beads were coated with the Emab and presented to ECAR modified T lymphocytes (**Fig. 2a** **)**. Binding of the Emab to the E-Tag should cross-link ECARs and thereby induce an activating signal in the modified T cell (**Fig. 2a** **)**. To compare the activation potential of such artificial, Emab decorated APCs, T cells modified with the PSCA-specific ECAR were further incubated with either commercially available polyclonal activator beads coated with mabs against the CD3 and CD28 antigen (αCD3/CD28 beads) or with target cells expressing the PSCA antigen on their surface. Only ECAR engrafted T lymphocytes were activated and released T cell-specific cytokines like interferon (IFN)-γ, tumor necrosis factor (TNF) and interleukin (IL)-2 in the presence of both Emab coated beads and antigen-expressing target cells (PC3-PSCA, **Fig. 2b**). In contrast, both ECAR engrafted T cells as well as T cells of the control groups released cytokines upon polyclonal stimulation with αCD3/CD28 beads (**Fig. 2b**). Moreover, ECAR engrafted T lymphocytes started to proliferate and expanded after activation with Emab coated beads or in the presence of PSCA expressing cells, whereas CAR negative T lymphocytes or T lymphocytes engrafted with the ECAR Stop did not or only marginally expand as a reaction to these stimuli (**Fig. 3a, b**). This observation could be verified for a series of 5 donors tested (**Fig. 3b**). Thus, the E-Tag incorporated into the scFv could be successfully used to trigger T cell activation and expansion independent of the CAR specificity by cross-linking CARs on the cell surface via the Emab coated beads.

Next, it was evaluated if ECAR engrafted human T lymphocytes can be expanded in large batches in the presence of exogenous cytokines as it would be required for a clinical application. Both PSCA- and CD33-specific ECAR engineered T lymphocytes could be expanded with Emab coated beads over a two week period in similar quantities as the control groups, which were non-specifically stimulated with αCD3/CD28 polyclonal activator beads (**Fig. 3c**). Upon stimulation with Emab coated beads the number of ECAR engrafted T cells increased on average 36-fold (range 10- to 95-fold), whereas T cells in the vector control group propagated on average 32-fold (range 5- to 90-fold) in the presence of αCD3/CD28 polyclonal activator beads. In the presence of Emab coated beads ECAR engrafted T lymphocytes expanded significantly better than non-modified T lymphocytes of the same batch (**Fig. 3c**). After the two-week expansion period the major portion of expanded T lymphocytes had a CD27^+^CD62L^+^ phenotype with no obvious differences between the Emab expanded, ECAR positive T lymphocytes and T lymphocytes of controls expanded with αCD3/CD28 polyclonal activator beads (**Fig. 3d**).

In conclusion, in comparison to polyclonal αCD3/CD28 activator beads magnetic beads coated with the Emab could be successfully used to expand ECAR engrafted T cells in similar or even higher numbers, but with the advantage that genetically modified T cells expanded preferentially and therefore could be enriched in the culture.

### ECAR Expressing T Cells can be Easily Purified via the Linker Epitope and Mediate Efficient *in vitro* and *in vivo* Anti-tumor Responses after Expansion

**Figure 4:**
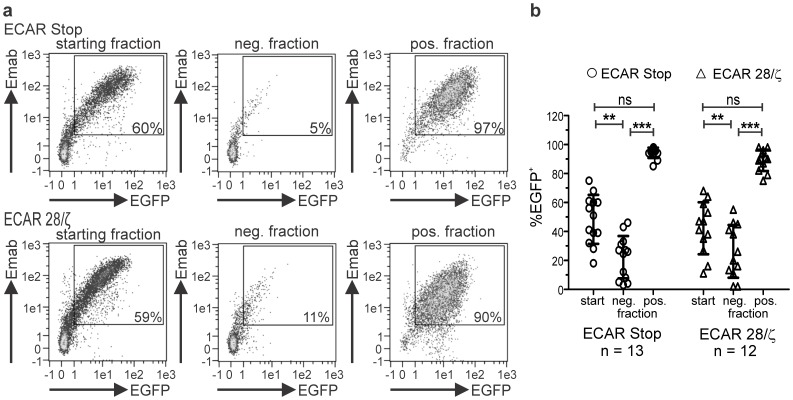
ECAR genetically modified human T cells can be purified via the CAR internal E-Tag. Expanded ECAR engrafted T lymphocytes were incubated with Emab coated magnetic beads and purified by magnetic cell sorting. Aliquots of expanded T lymphocytes before the purification procedure (starting population), of the flow-through (neg. fraction) and the eluted ECAR bearing fraction (pos. fraction) were subsequently stained for ECAR surface expression using the E-Tag specific mab. Staining results for one representative donor (**a**) and pooled results for 12 (ECAR 28/ζ) and 13 (ECAR Stop) donors are shown (**b**). Statistical analysis was performed using non-parametric one-way ANOVA (Kruskal-Wallis test) and post-hoc Dunn’s Multiple Comparison test (**p<0.01, ***p<0.001).

For a clinical setting the adoptive transfer of pure populations of CAR modified T lymphocytes not contaminated with non-modified T lymphocytes would be beneficial to obtain and define clear dose-related effects in treated patients and to reduce unwanted side effects due to cytokine release from non-modified but non-specifically activated co-transferred T cells. Therefore, we tested if it is possible to purify ECAR modified T lymphocytes from still mixed populations after expansion. Using commercially available magnetic beads coated with the Emab, ECAR modified T lymphocytes could be purified by passing the cells over magnetic columns. The isolation procedure resulted in a purity of up to 98% pure ECAR modified T cell populations with only marginal loss of CAR modified cells in the flow-through (CAR negative) fraction (**Fig. 4a**) and could be consistently repeated for several donors irrespective of the CAR specificity used to modify T lymphocytes (**Fig. 4b**)**.**

**Figure 5:**
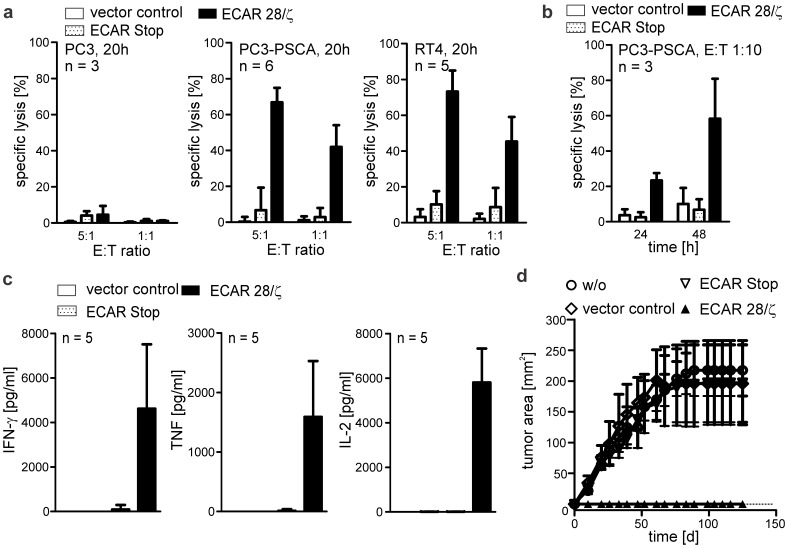
Expanded PSCA-specific ECAR engrafted T lymphocytes efficiently mediate anti-tumor effects both *in vitro* and *in vivo*. (**a,b**) Cytotoxicity assay with expanded and purified PSCA-specific ECAR modified T lymphocytes and two different PSCA-positive tumor cell lines (PC3-PSCA, RT4). Parental PSCA-negative PC3 tumor cells were used as control. ECAR modified or empty vector modified T cells were incubated with ^51^Cr-labeled target cells at different effector to target cell (E:T) ratios for indicated time periods. Mean lysis ± SD is shown for several individual donors as indicated. (**c**) Release of proinflammatory and T cell specific cytokines from PSCA-specific ECAR modified T lymphocytes upon incubation with PSCA-positive target cells. T lymphocytes were incubated with 2*10^4^ target cells at an E:T ratio of 5∶1 for 20h. Cell-free supernatant taken from cultures was analyzed with commercially available ELISA kits. (**d**) NMRI (nu/nu) mice were subcutaneously injected with 1.8*10^6^ tumor cells (PC3-PSCA) alone (w/o), or with the same number of T lymphocytes either modified with an empty vector control (vector control), with an ECAR without signaling subunit (ECAR Stop) or a PSCA-specific ECAR with signaling subunit (ECAR 28/ζ). Developing tumors were quantitatively measured using a caliper and mean tumor sizes ± SD were plotted versus time for each group.

**Figure 6:**
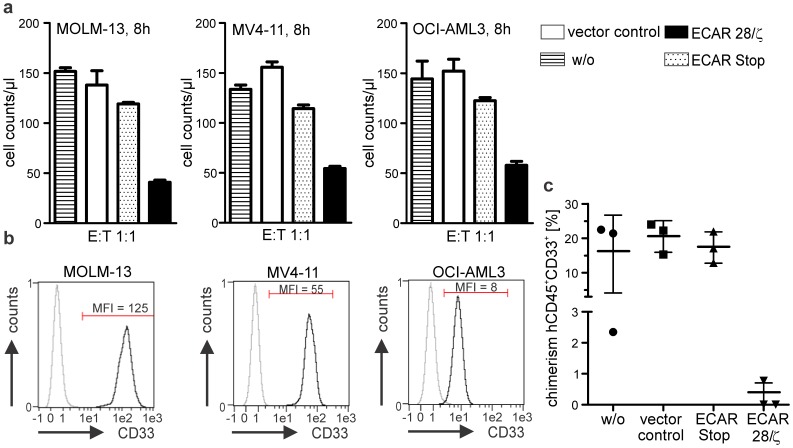
Expanded CD33-specific ECAR modified T lymphocytes kill CD33-positive AML cell lines *in vitro* and prevent the establishment of AML xenografts *in vivo*. (**a**) Flow-cytometry based cytotoxicity assay with expanded and purified CD33-specific ECAR modified T lymphocytes and different AML cell lines. T lymphocytes were cultured with 3*10^4^ proliferation dye eFluor670 labeled AML cells at an E:T ratio of 1∶1. After 8h living AML cells were quantitatively determined using a MACSQuant Analyzer. Results for one representative donor out of five (MOLM-13) or three (OCI-AML3, MV4-11) are shown. (**b**) AML cell lines used for experiments express varying densities of CD33 on their cell surface. Cells were stained with a PE-conjugated anti-CD33 mab and analyzed for antigen expression using a MACSQuant Analyzer. (**c**) 1*10^5^ MOLM-13 AML cells were cultured alone (w/o), or together with T lymphocytes either modified with an empty vector control (vector control), with an ECAR without signaling subunit (ECAR Stop) or a CD33-specific ECAR with signaling subunit (ECAR 28/ζ) at an E:T ratio of 5∶1 for 4 h and administered to NOD/SCID IL2Rγ^−/−^ mice by retrobulbar injection. Due to eye tumor development in the control groups, mice had to be sacrificed fourteen days later and bone marrow chimerism was analyzed using a flow cytometer (BD LSRII). Percentage of human CD45^+^/CD33^+^ double positive cells are plotted for each individual mouse. T lymphocytes were irradiated at 5 Gy before starting the culture to prevent xenograft reactions in the recipient mice.

Finally, the expanded and purified T lymphocytes were tested for their *in vitro* and *in vivo* tumor cell killing abilities. PSCA-specific ECAR modified T cells from several human donors were successfully redirected to two PSCA expressing tumor cell lines and lysed target cells upon incubation, whereas PSCA negative PC3 cells were not attacked (**Fig. 5a**). Even at very low effector to target cell (E:T) ratios, ECAR modified T lymphocytes were able to lyse PSCA-positive tumor cells over a prolonged time period **(** **Fig. 5b** **)**. This observation demonstrates that the modified T lymphocytes are capable of serial killing of target cells, an important point in regard to a clinical application in case that only few modified T lymphocytes reach the site of tumor growth. Upon incubation with PSCA-positive target cells expanded and purified T lymphocytes harboring a PSCA-specific ECAR with signaling subunit (ECAR 28/ζ) started to secrete high amounts of cytokines, whereas T lymphocytes modified with an empty vector control or an ECAR Stop did not release any cytokines (**Fig. 5c**). In a mouse model, ECAR engrafted T cells completely prevented the establishment and outgrowth of human xenograft tumors subcutaneously injected into nude mice (**Fig. 5d** **).** No tumor development was observed for treated mice until the experiment was finally stopped after 130 days and all animals of this group survived, whereas the last remaining animal of the control groups had to be sacrificed on day 83 due to excessive tumor growth **(Fig. S1c**). In a similar way, CD33-specific ECAR modified T lymphocytes proved to efficiently eradicate AML cell lines *in vitro* independent of the antigen surface density (**Fig. 6a, b**) and to release T cell cytokines upon encounter of target tumor cells similar to T cells engrafted with PSCA specific ECAR (**data not shown**). In addition, the establishment of AML cells in the bone marrow of NSG mice could be prevented by CD33-specific ECAR modified T lymphocytes (**Fig. 6c** **).** In contrast to the control groups none of the mice of the treatment group developed tumors at the injection site **(Fig. S1d)** and bone marrow chimerism for human CD45^+^CD33^+^ double positive cells was below 1% in all treated animals, whereas in the control groups chimerism exceeded on average 15% of total bone marrow cellularity (**Fig. 6c** **)**.

## Discussion

After years of intensive research the CAR technology seems now to be ready to close the gap between bench and bedside for tumor treatment. Encouraging clinical responses were observed in recently published clinical trials, in which patient-derived T cells were armed with CD19 specific CARs for treatment of malignancies of B cell origin [16]–[19]. However, the success of therapy depends in particular on the availability of sufficient numbers of CAR modified T cells for adoptive transfer into the patient. So far, different methods have been evaluated to expand antigen-specific T cells *in vitro* before retransfer, but either the procedures are non-specific or can only be applied for a certain therapeutical setting as in case of antigen-expressing expander cell lines [20]–[23]. Therefore we decided to develop and evaluate a universal technique which allows expansion and purification of CAR modified lymphocytes independent of antigen-specificity of CAR binding domains. The incorporation of a small epitope into the linker between the heavy and light chain of both scFvs tested in this study does not harm functionality of the CAR binding moiety as demonstrated by our *in vitro* and *in vivo* experiments. Most importantly, an activating signal can exclusively be induced in the CAR modified T cells by adding magnetic beads coated with an epitope specific mab ultimately leading to proliferation and expansion of just the genetically modified T cells. The observed expansion rates and resulting phenotype of expanded signaling ECAR engineered T cells after two expansion cycles with the Emab coated beads is similar to the comparative cultures of non-specific T cells of the same donors expanded with commercially available T cell activator beads. This observation is in line with the fact, that similar signaling cascades were induced in both systems. On the one hand, endogenous TCR/CD3 complexes and the costimulatory CD28 molecules are cross-linked by the antibodies coated onto the non-specific activator beads, and, on the other hand, the combined CD28 and CD3zeta signaling subunits as part of the ECAR are triggered by the Emab coated beads. It will be of interest to apply the E-Tag expansion system to T cells engrafted with 3^rd^ generation ECAR constructs, which include an additional second costimulatory domain e.g. from tumor necrosis factor receptors like 4-1BB (CD137) or Ox40 (CD134) [34], [35]. Our here presented results give reason to assume that expansion rates of T cells modified with 3^rd^ generation ECARs should be even superior to expansion rates induced by conventional CD3/CD28 activator beads. Nevertheless, a second-generation CD28/CD3ζ CAR was used in the present study on purpose as it is highly similar in its basic structure to those CD28/CD3ζ CAR constructs tested in recent clinical trials to allow for comparison with published data [36]. In this way, our observed expansion rates for ECARs activated with Emab coated beads are comparable to published mean fold expansion rates of CAR modified, patient-derived T cells with anti-CD3/anti-CD28 coated activator beads over a comparable time period [16]. However, in case of Emab bead mediated expansion only T cells engrafted with the ECAR profoundly proliferate leading to a significant enrichment of CAR modified T cells in the culture. According to published data on recent clinical trials with CD19-specific CAR constructs on average 28% (range 4–70%) of infused T cells were CAR positive after *in vitro* modification and expansion [16], [17]. In these trials, retro- or lentiviral vector systems were used for genetic modification of patient derived T cells, which represent the currently most reliable systems for gene transfer [36]. Due to specific expansion of CAR expressing T cells using our novel ECAR system clinically relevant numbers of highly pure T cells could be obtained even from patient cell samples with initially low transduction efficiency. Moreover, applying our specific ECAR system would also allow for reduction of lenti- or retroviral titers applied to T cell cultures for genetic modification. Adding lower viral titers will consequently result in less insertion events per cell and therefore minimize the risk of insertional mutagenesis [37], an important point especially in case hematopoietic or induced stem cells are genetically modified [38], [39].

Besides, not only the number of genetically modified T cells after *ex vivo* expansion, but also the phenotype of the generated T cell batch will determine the outcome of cellular treatment. The functional characterization demonstrates that after *ex vivo* expansion with Emab coated beads the ECAR modified T cells are able to kill antigen-expressing cells very efficiently, whereas antigen-negative target cells are not attacked. In a similar way, ECAR engrafted T cells secrete high amounts of proinflammatory and T cell specific cytokines upon incubation with antigen-expressing cells, proving that the expanded cells are fully functional. *In vitro* observations were confirmed by *in vivo* experiments, which demonstrate that the establishment of PCa or AML xenografts is prevented by ECAR modified T cells. Interestingly, concerning the AML mouse model only in mice injected with the signaling ECAR about half of human CD45^+^ cells detected in the bone marrow are T cells, whereas in the two other groups treated with vector control or ECAR Stop modified T cells percentage of CD3^+^ cells is in the range of background noise observed in the control group treated only with AML cells but without T cells (**Fig. S1e**). Obviously, signaling by the ECAR upon antigen encounter does not only induce killing of AML cells, but also improves the survival of CAR modified cells. This observation is in line with published data from other groups which treated leukemias with CAR modified T cells in mouse models [40]. In addition, *in vitro* experiments prove that ECAR modified T cells exhibit profound proliferation and expansion over several days after antigen encounter in the absence of exogenous cytokines, whereas control T cells either modified with vector control or ECAR Stop constructs nearly vanish under the same conditions (**Fig. S1f**). In this context the predominantly CD62L^+^ CD27^+^ double-positive phenotype of ECAR modified T cells after expansion culture is of interest, as T lymphocytes expressing both surface markers have been described as central memory cells which are able to induce long-term anti-tumor responses in hosts [41], [42].

Taken together, the incorporation of the E-Tag as a linker into the scFv binding moiety of CARs does not hinder surface expression and functionality of the newly constructed ECARs in human T lymphocytes. As demonstrated, the epitope can be used to clearly identify, expand and purify ECAR engineered T lymphocytes using a mab specific for the E-Tag. Our work is in line with the recently published work by Wang and colleagues [43]. In their study a truncated form of human epidermal growth factor receptor (EGFRt) was co-expressed with a CAR and an anti-EGFR ab was used to enrich genetically engineered T cells. However, a specific expansion via CAR triggering was not possible by this approach [43]. In the same study it was demonstrated, that *in vivo* tracking and ablation of engineered T cells can be performed with mabs targeting the extracellular domain of EGFRt [43]. Although we have not performed such experiments so far, in a similar way Natural Killer (NK) cell activating derivatives of the Emab might be used for *in vivo* elimination of ECAR modified T cells if necessary. As expansion with mab coated beads and purification via magnetic columns are techniques, which have already been established under good manufacturing practice for cellular immunotherapy, the clinical application of the herein described technique should be straight forward.

Most importantly, from our data one can expect that the E-Tag can be incorporated into any scFv or other binding moiety used for CAR construction without hampering their function. Therefore, the technique can most probably be applied universally for all CAR modified T lymphocytes independent of their target specificity. As scFvs and the variable domains of TCRs have in principle the same architecture, the E-Tag might also be useful for expansion and purification of T cells genetically modified with transgenic TCRs. The ECAR technique offers a reasonable solution to obtain sufficient numbers of highly pure CAR engrafted T lymphocytes. For a long time the lack of truly tumor-specific antigens has limited the application of genetically engineered T lymphocytes to certain cancer types. Recently it has been demonstrated, that the risk of unwanted “on target, off side” effects in case target antigens are not exclusively restricted to tumor tissues can be circumvented by balanced combination of activating and costimulatory CARs with independent antigen-specificities [44]. Thus, the door is now open to target a broad spectrum of different cancers lacking exclusively tumor-specific antigens with CAR modified T cells in future. The application of well-defined (E-Tag) engineered T cell populations in clinical trials would help to determine minimal cell doses necessary to achieve objective anti-tumor responses and might help to improve overall therapeutic efficiency of genetically engineered T lymphocytes approaches.

## Materials and Methods

### Ethics Statement

Human peripheral blood mononuclear cells (PBMCs) were isolated either from buffy coats supplied by the German Red Cross (Dresden, Germany) or from fresh blood of healthy donors with their written consent. The study including the consent form was approved by the local ethics committee of the university hospital of the medical faculty of Carl-Gustav-Carus TU-Dresden (EK27022006). All animal experiments were performed according to the German animal protection law with permission from the responsible local authorities and ethics committee (Sächsische Landesdirektion, 24-9168.11-1/2013-32).

### Isolation and Cultivation of T Cells

The isolation of human T cells from PBMC was performed as recently described [45], using either a Pan T cell Isolation Kit (Miltenyi Biotec GmbH, Bergisch Gladbach, Germany) or the PluriSelect CD3^+^ Isolation Kit (PluriSelect GmbH, Leipzig, Germany). T cells were cultured in RPMI 1640 complete medium supplemented with 200 U/ml IL-2 (Proleukin S, Novartis Pharmaceuticals, Horsham, UK), 5 ng/ml IL-7 and 5 ng/ml IL-15 (ImmunoTools, Friesoythe, Germany) at 37°C in a humidified atmosphere of 5% CO_2_ at densities of 1−2*10^6^ cells/ml. T cells were starved in complete RPMI 1640 without recombinant cytokines 24h before experiments were performed.

### Cell Lines

The PSCA-negative prostate carcinoma cell line PC3 wild type (wt), the PC3-PSCA cell line, which was genetically modified to permanently express PSCA [45], RT4, MOLM-13 and MV4-11 were cultured in RPMI 1640 complete medium. Human embryonic kidney (HEK) 293T cells and OCI-AML3 were cultured in DMEM complete medium. Cell lines (HEK)293T, PC3, and RT4 were purchased from the American Type Culture Collection (ATCC), MOLM-13 (ACC-554), MV4-11 (ACC-102) and OCI-AML3 (ACC-582) were purchased from the Leibniz Institute German Collection of Microorganisms and Cell Cultures (DSMZ). Cells were maintained at 37°C in a humidified atmosphere of 5% CO_2_ [32], [33].

### Construction of PSCA- and CD33-specific CARs

The cloning of the PSCA-specific scFv 7F5 [29], [45] as well as the generation and cloning of the CD33-specific scFv DRB2 [33], [46] were described recently in detail. Both scFvs were further modified by exchanging the common GS-linker between the variable heavy and light chain against a new unique linker element. Incorporation of this linker element that consists of two G_4_S_1_ elements at the 5′ and 3′ end and in between a peptide sequence of 10 aa (KPLPEVTDEY) from the human La/SS-B protein (E-Tag) was performed by overlap PCR using primers coding for the new linker sequence. A mab termed 5B9 generated by standard hybridoma fusion technique was identified to be reactive against this epitope sequence [25]. The CD28 and the CD3ζ domain of the ECARs were amplified from cDNA isolated from human PBMCs. The CD28 domain was amplified from aa 22–220 using specific primers and fused to the intracellular chain of CD3ζ amplified from aa 55–220 by PCR. Between the CD28 signaling subunit and the CD3ζ chain a 4×G_4_S_1_ linker was introduced. Finally, αPSCA or αCD33 scFv was fused at the 5′ end of the CD28 coding sequence with an additional G_4_S_1_ linker in between the scFv and CD28. As a control, an ECAR without an intracellular signaling chain was constructed. For that purpose, CD28 was amplified by PCR from aa 20–185, which excludes the intracellular signaling domain. All ECAR constructs were C-terminally fused to an EGFP marker protein separated by a 2pA protease site derived from the *Thosea asigna* virus, which allows an independent translation of ECAR and EGFP from a single mRNA in modified T cells [47].

### Lentiviral Vector Construction and Genetic Modification of Human T Cells

The lentiviral transfer vector p6NST60 is a derivative of the recently described p6NST90, containing a spleen focus forming virus (SFFV) U3 promoter instead of the human ubiquitin C promoter driven EGFP expression cassette [48]. The EGFP expression cassette was exchanged for a multiple cloning site (MCS) containing unique *XbaI* and *HpaI* sites. The open reading frames (*orf*) of the ECAR-EGFP expression cassette with or without intracellular signaling domain were cloned into the mentioned restriction sites of the MCS using *NheI* and *PmeI* restriction sites at the 5′ and 3′ end of the ECAR-EGFP *orf*, respectively. To generate Vesicular stomatitis virus G glycoprotein (VSV-G) pseudotyped lentivirus, the lentiviral vector DNA was co-transfected into HEK293T cells by using linear polyethylenimine (Polysciences Europe GmbH, Eppelheim, Germany) together with lentiviral packaging plasmid pCD/NL-BH and VSV-G encoding pMD-GM plasmid. Viral supernatants were harvested, concentrated by ultra centrifugation and stored at −80°C as described elsewhere [30], [48], [49]. Viral titers were determined by limiting dilution transduction on HT1080 cells as described elsewhere [48], [49].

### Cytotoxicity Assay

The cytotoxicity of PSCA-specific ECAR engrafted T cells was analyzed in a ^51^Cr release assay as described previously [31]–[33]. Briefly, effector T cells were cocultured with 5*10^3^ ^51^Cr labeled tumor cells at different E:T ratios for indicated time periods. Released ^51^Cr was determined with the help of a beta counter (PerkinElmer Life Sciences, Rodgau-Rügesheim, Germany). Killing of AML cells by CD33-specific ECAR engrafted T cells was in addition determined in a flow cytometry based cytotoxicity assay as described elsewhere [31]–[33]. To distinguish effector from target cells, the latter were stained with cell proliferation dye eFluor670 (eBioscience, San Diego, USA). Effector and labeled target cells were incubated together for the indicated time periods and at indicated E:T ratios. Subsequently absolute living cell numbers were quantified. Therefore, an aliquot of at least 1/10 of total sample volume was taken at indicated time points and analyzed using a MACSQuant Analyzer and MACSQuantify software (Miltenyi Biotec).

### ECAR Engrafted T Cell Expansion Assay and Cytokine ELISA in the Absence of Recombinant Cytokines

To compare different activation stimuli 1*10^5^ modified or control T cells were seeded in 96-well plates in triplets and either Emab coated beads, αCD3/CD28 coated polyclonal activator beads or PSCA expressing PC3 cells were added to T cell cultures at a 1∶4 ratio. Absolute cell numbers of triplets were quantified at day 0 and at indicated time points. To determine cytokine release, cells were spun down and supernatants were collected. The cytokine concentration in the supernatants was determined by using the OptEIA™ Human IFN-γ, OptEIA™ Human IL-2 and OptEIA™ Human TNF ELISA Kits (BD Biosciences, Heidelberg, Germany). Experiments with expanded and purified ECAR T cells to determine cytokine release in the presence of tumor target cells were performed in the same way. For expansion experiments with expanded and purified ECAR T cells similar numbers of T cells and eFluor670 (eBioscience, San Diego, USA) stained target cells were seeded in 96-well plates in triplets in culture medium without the addition of exogenous cytokines and absolute cell numbers of triplets were quantified at day 0 and indicated time points from an aliquot (at least 1/10 of total sample volume) using a MACSQuant Analyzer.

### ECAR Engrafted T Cell Expansion Assay and Purification

ECAR engrafted T cells and control samples were seeded in complete RPMI 1640 supplemented with cytokines at densities of approximately 1*10^6^ cells/ml in 24-well plates. For expansion either Emab coated beads or αCD3/CD28 coated polyclonal activator beads were added to T cell cultures at a 1∶4 ratio. Absolute cell numbers were determined at day 0 and before as well as after bead removal and/or media exchange over 14 days. ECAR engrafted T cells were distinguished from ECAR negative T cells by EGFP marker. ECAR armed T cells were purified with Emab coated anti-mouse IgG microbeads (Miltenyi Biotec) on MS MACS Columns (Miltenyi Biotec) according to the manufactures protocol.

### Flow-cytometry Analysis

Isolated T cells were stained with fluorochrome-labeled mabs directed against human CD4/VioBlue (Miltenyi Biotec, clone VIT4), CD3/PE-Cy7 (Biolegend, San Diego, USA, clone UCHT1), CD8/APC (BD Bioscience, clone RPA-T8), CD27/PE (BD Bioscience, clone M-T271) and CD62L/PacificBlue (Biolegend, clone DREG-56). For detection of ECAR surface expression, T cells were incubated with anti-La mab 5B9 [16] and subsequently stained with PE-labeled goat anti-mouse IgG (Beckmann Coulter, Krefeld, Germany). Samples were analyzed using the MACSQuant Analyzer and the MACSQuantify software (Miltenyi Biotec). In order to assess expansion rates of ECAR armed T cells, absolute T cell numbers were quantified using a MACSQuant Analyzer and MACSQuantify software (Miltenyi Biotec) as described elsewhere [25].

### Mouse Models

Mice were kept under standardized environmental conditions and received autoclaved food, water, and bedding.

#### NMRI (nu/nu) mouse subcutaneous xenograft tumor model

Eight-week old NMRI (nu/nu) mice were subcutaneously transplanted with 1.8*10^6^ PSCA-expressing PC3 cells (5 mice/group) alone or together with either purified ECAR modified T cells or control vector modified T cells at an E:T ratio of 1∶1. Tumor growth was recorded by measuring width and length of palpable tumors and tumor area was calculated. If tumor size exceeded 18mm in one direction, mice were sacrificed due to ethical reasons.

#### NOD/SCID IL2Rγ^−/−^ mouse AML bone marrow xenograft model

Eight- to ten-week-old NOD/SCID IL2Rγ^−/−^ mice were intravenously injected with 1*10^5^ MOLM-13 cells. Beforehand, AML cells were cultured alone or in the presence of either purified ECAR or control vector modified T cells at an E:T ratio of 5∶1 for 4 h. Prior to the experiment, T cells were irradiated at 5 Gy to prevent xenograft reaction in recipient mice [50]. Mice were sacrificed when visible tumors developed at injection site and single-cell suspensions from bone marrow obtained from femur and tibia of the left hind leg were prepared. Erythrocytes were removed by lysis and nucleated cells were stained with anti-mouse CD45.1/PE-Cy7 (eBioscience, clone A20), anti-human CD3/APC-eFluor780 (eBioscience, clone SK7), CD19/APC (BD Bioscience, clone HIB19), CD33/PE (eBiosience, clone HIM3-4), and CD45/AlexaFluor700 (Biolegend, clone HI30) mabs. Doublet discrimination was routinely carried out and dead cells were excluded by 4, 6 diamidino-2-phenylindole (DAPI)-staining (Sigma-Aldrich, Taufkirchen, Germany). All measurements were performed on a BD LSRII FACS machine (BD Biosciences). Data analysis was realized using FlowJo-software (Tree Star Inc., Ashland, USA).

### Statistical Analysis

Statistical analysis was performed with GrapPad Prism software version 5.0 (GraphPad Software Inc., La Jolla, CA, USA) using non-parametric Mann-Whitney test for two samples or one-way ANOVA (Kruskal-Wallis test) and post-hoc Dunn’s Multiple Comparison test for multiple samples.

## Supporting Information

Figure S1 **ECAR genetically modified human T cells prevent the establishment of human xenograft tumors** ***in vivo*** **.** (**a**) Binding affinity of the PSCA-specific scFv with internal E-Tag linker was compared to the same scFv having an internal glycin/serine linker by means of titration assay on PSCA-expressing PC3 cells. Bound scFv molecules were detected using a PE-labeled antibody directed against the C-terminal His tag (Miltenyi, clone GG11-8F3.5.1) (**b**) Following lentiviral gene transfer human T cells were stained for the presence of CD33-specific ECARs on the cell surface using the Emab. Surface expression of ECARs as well as co-translational EGFP expression in the cytoplasm could be detected. (**c**) Survival curve of mice subcutaneously injected with human PCa xenografts. NMRI (nu/nu) mice were subcutaneously injected with 1.8*10^6^ tumor cells (PC3-PSCA) alone (w/o), or with the same number of T lymphocytes either modified with an empty vector control (vector control), with an ECAR without signaling subunit (ECAR Stop) or a PSCA-specific ECAR with signaling subunit (ECAR 28/ζ). For ethical reasons animals were sacrificed as soon as the developing tumor exceeded a length of 18 mm in one direction. (**d,** **e**) Development of local tumors at the injection site in mice with AML xenografts. 1*10^5^ MOLM-13 AML cells were cultured alone (w/o), or together with T lymphocytes either modified with an empty vector control (vector control), with an ECAR without signaling subunit (ECAR Stop) or a CD33-specific ECAR with signaling subunit (ECAR 28/ζ) at an E:T ratio of 5∶1 for 4 h and administered to NOD/SCID IL2Rγ^−/−^ mice by retrobulbar injection. The percentage of animals with clearly visible tumors at the end of the experiment is given (**d**) and percentage of human CD45^+^/CD3^+^ double positive cells among human CD45^+^ in the bone marrow is plotted for each individual mouse (**e**). (**f**) Expansion of CD33-specific ECAR engrafted T cells upon encounter with CD33 expressing MOLM-13 AML cells. 3*10^4^ MOLM-13 AML cells were cultured together with T cells either modified with an empty vector control (vector control), with an ECAR without signaling subunit (ECAR Stop) or a CD33-specific ECAR with signaling subunit (ECAR 28/ζ) at an E:T ratio of 1∶1 for 6 days. Mean and standard deviation of triplicates are given for one representative donor out of three analyzed.
(TIF)
