# Supplementary figures and images for: A Novel Ex Vivo Isolation and Expansion Procedure for Chimeric Antigen Receptor Engrafted Human T Cells
Source: PLoS One. 2014 Apr 3;9(4):e93745. doi: 10.1371/journal.pone.0093745 (PMC3974878; doi:10.1371/journal.pone.0093745)

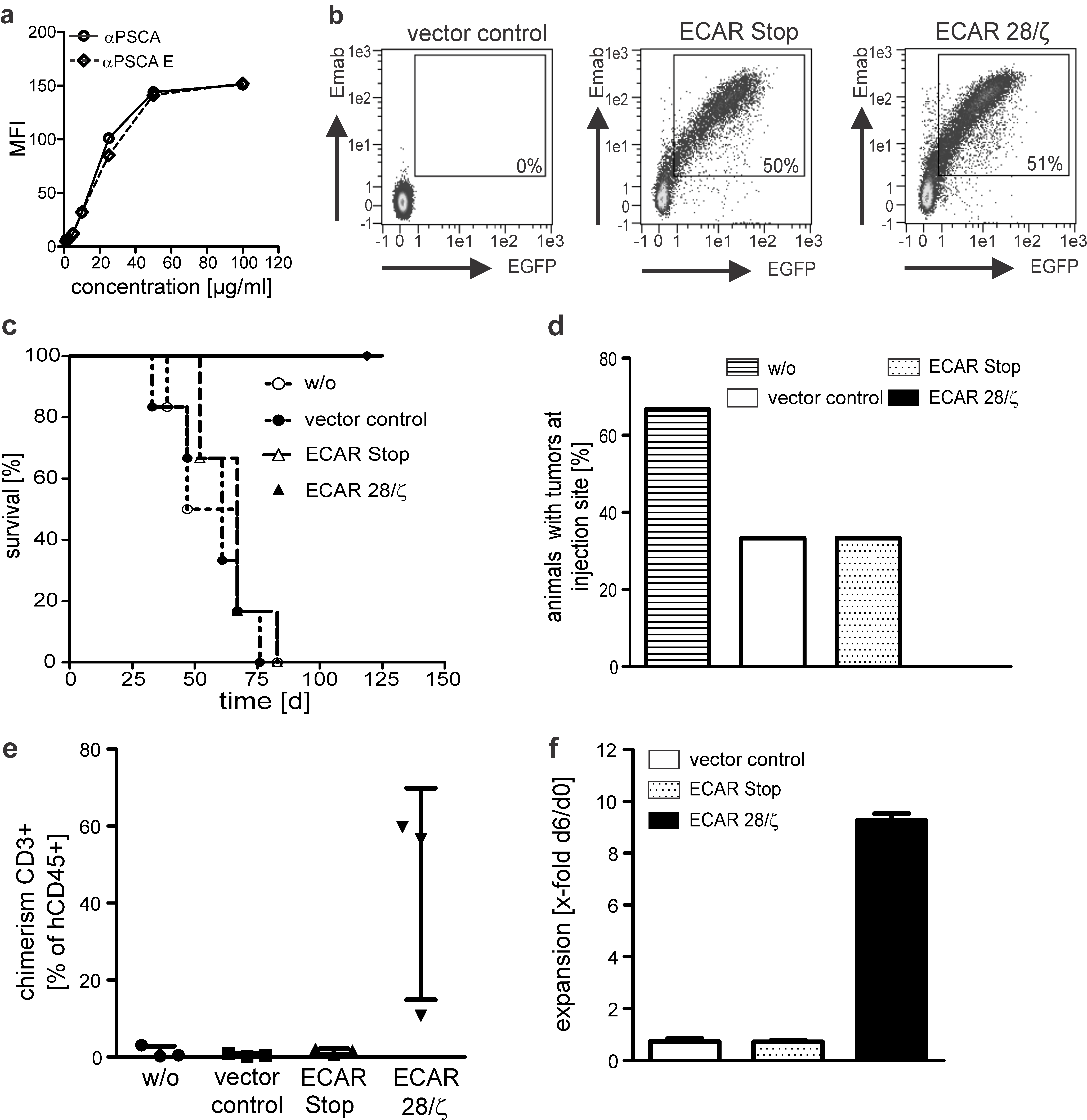

Supplement: Figure S1 — ECAR genetically modified human T cells prevent the establishment of human xenograft tumors in vivo . (a) Binding affinity of the PSCA-specific scFv with internal E-Tag linker was compared to the same scFv having an internal glycin/serine linker by means of titration assay on PSCA-expressing PC3 cells. Bound scFv molecules were detected using a PE-labeled antibody directed against the C-terminal His tag (Miltenyi, clone GG11-8F3.5.1) (b) Following lentiviral gene transfer human T cells were stained for the presence of CD33-specific ECARs on the cell surface using the Emab. Surface expression of ECARs as well as co-translational EGFP expression in the cytoplasm could be detected. (c) Survival curve of mice subcutaneously injected with human PCa xenografts. NMRI (nu/nu) mice were subcutaneously injected with 1.8*106 tumor cells (PC3-PSCA) alone (w/o), or with the same number of T lymphocytes either modified with an empty vector control (vector control), with an ECAR without signaling subunit (ECAR Stop) or a PSCA-specific ECAR with signaling subunit (ECAR 28/ζ). For ethical reasons animals were sacrificed as soon as the developing tumor exceeded a length of 18 mm in one direction. (d, e) Development of local tumors at the injection site in mice with AML xenografts. 1*105 MOLM-13 AML cells were cultured alone (w/o), or together with T lymphocytes either modified with an empty vector control (vector control), with an ECAR without signaling subunit (ECAR Stop) or a CD33-specific ECAR with signaling subunit (ECAR 28/ζ) at an E:T ratio of 5∶1 for 4 h and administered to NOD/SCID IL2Rγ−/− mice by retrobulbar injection. The percentage of animals with clearly visible tumors at the end of the experiment is given (d) and percentage of human CD45+/CD3+ double positive cells among human CD45+ in the bone marrow is plotted for each individual mouse (e). (f) Expansion of CD33-specific ECAR engrafted T cells upon encounter with CD33 expressing MOLM-13 AML cells. 3*104 MOLM-13 AML ce [file pone.0093745.s001.tif]
